# Latent Diffusion Process With Mechanistic Guidance For Designing Functionally Graded Metamaterials With Perfect Connectivity

**DOI:** 10.1002/advs.76914

**Published:** 2026-07-30

**Authors:** Jongbin Yu, Dosung Lee, Namjung Kim

**Affiliations:** ^1^ Department of Mechanical Engineering Gachon University Seongnam South Korea; ^2^ Department of Mechanical Engineering Sogang University Seoul South Korea

**Keywords:** deep generative model, discrete latent space, functionally graded metamaterials, graph‐based metamaterial, inverse design, latent diffusion process

## Abstract

Recent advancements in artificial intelligence (AI)–based design strategies have expanded the ability to generate complex mechanical metamaterials across multiple length scales. However, achieving precise control of mechanical properties while preserving structural connectivity remains a major challenge, especially for functionally graded metamaterials with heterogeneous unit cell architectures. Here, a latent diffusion–based design framework is proposed for 3D graph metamaterials that enables stable generation and accurate inverse design in a discrete, topology‐aware latent space. By integrating vector‐quantized latent representations with a diffusion‐based generative process and mechanistic guidance, the framework effectively explores complex design spaces while steering generated structures toward target elastic properties. The proposed approach enables the generation of graph metamaterials ranging from repetitive lattices to functionally graded architectures with smooth mechanical transitions and robust connectivity. These results demonstrate that latent diffusion with mechanistic guidance provides a scalable alternative to conventional interpolation‐based or purely data‐driven generative models for mechanistically optimized metamaterial design.

## Graphical abstract

A latent diffusion‐based framework is proposed for designing functionally graded metamaterials with perfect connectivity. By integrating vector‐quantized latent representations with mechanistic guidance, the framework enables accurate inverse design toward target elastic properties. A hybrid interpolation strategy combining latent interpolation and diffusion‐based guidance achieves smooth mechanical transitions while preserving complete connectivity, as demonstrated through a biomedical femoral implant application.

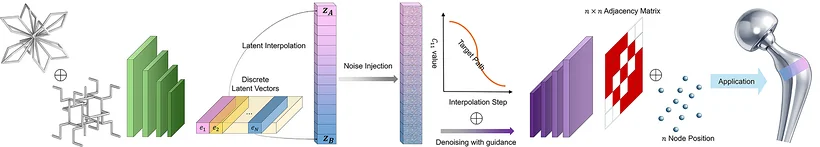

## 1 Introduction

Functionally graded metamaterials (FGMs) are architected materials that enable continuous transitions in mechanical properties tailored to specific applications through spatially varying microstructures. By gradually modulating attributes such as density and stiffness, FGMs overcome fundamental limitations of homogeneous materials, particularly in applications where abrupt property discontinuities can lead to stress concentrations or mechanical failure [1, 2, 3]. Owing to this capability, FGMs have attracted significant attention across a broad range of engineering applications, including energy‐absorbing structures [4, 5], resisting thermal stresses in demanding environments [6], manipulating elastic wave propagation [7], and adaptive or robotic systems requiring programmable mechanical responses [8], with recent demonstrations including 4D‐printed bioinspired metamaterials that exhibit tunable mechanical behavior [9].

The effectiveness of such gradient‐based design strategies is further supported by natural material systems, where spatial and hierarchical variations in composition and structure are commonly employed to achieve multifunctionality. Representative examples include sea urchin spines, bamboo, mollusk shells, and human bone, which achieve high strength, damage tolerance, and functional integration across multiple length scales through naturally evolved gradients [10, 11, 12, 13]. Inspired by these biological paradigms, extensive research efforts have been devoted to developing FGMs that provide spatially continuous mechanical responses while maintaining structural integrity, with particular emphasis on biomedical implants where mechanical compatibility with surrounding tissues is essential [14, 15, 16].

Despite these advances, the design of FGMs with precise control over graded microstructures remains a significant challenge. Traditional computational approaches, such as finite element method (FEM)‐based optimization [17] and rule‐of‐mixtures methods [18], Mori‐Tanaka scheme [19], generally depend on prior assumptions regarding microstructural topology, grading profiles, or parameterized design spaces. More recent computational strategies extend graded design to geometrically complex domains, such as conformal filling gradient lattice structures generated by multiscale isogeometric topology optimization [20], yet they likewise rely on predefined parametric or optimization‐based formulations. While FEM‐based optimization can be computationally demanding, homogenization approaches may be limited in their ability to capture complex graded architectures. Another common strategy for achieving functionally graded properties is to vary internal features such as cell size, density, shape, and wall thickness [21, 22]. This approach, while effective in some cases, faces significant limitations in navigating the high‐dimensional, nonlinear, and often multimodal design spaces of FGMs, particularly as the complexity of systems increases. Recent advancements in artificial intelligence (AI) offer transformative solutions to these challenges. AI‐based design strategies, including generative models such as variational autoencoders (VAEs) [23], generative adversarial networks (GANs), and their derivatives [24, 25, 26] enable the efficient exploration of the design space and produce complex microstructural configurations. By automating feature extraction and leveraging data‐driven decision‐making, these models significantly enhance the design process for FGMs. This capability has opened new opportunities across diverse industries, including automotive [27], aerospace [28], biomedical [29], and energy [30], where tailored material properties are critical for innovation and performance.

Among these generative techniques, latent space‐based strategies have attracted particular attention, as they enable inverse design and exploration of structure–property relationships by embedding high‐dimensional microstructures into compact representations [31, 32]. Recent advances have expanded the applications of latent space‐based design strategies, introducing frameworks that accommodate structural complexity and model both linear and nonlinear behaviors [33, 34]. Researchers are leveraging latent space to tackle challenges such as inverse design of nonlinear metamaterials composed of single [35] or multi‐materials [36], development of size‐agnostic models for inverse tasks [37], and dynamic modulation of metamaterial responses [38]. These advances have expanded the design space of multi‐functional and highly optimized structures, accelerating the discovery of achievable configurations. In particular, graph‐based latent representations have recently enabled compact encoding of 3D lattices and the generation of new unit cells through arithmetic operations in latent space [39], graph‐algorithmic synthesis of irregular metamaterials with guaranteed interconnectivity [40], and diffusion‐based inverse design of curved truss structures within a continuous latent space [41]. Nevertheless, these studies have largely focused on individual or repeatable unit cells rather than the spatially continuous assembly of heterogeneous cells required for functionally graded metamaterials, where interface compatibility and smooth property transitions must be achieved simultaneously. As a result, connectivity between dissimilar microstructures is often handled through ad hoc strategies, which reduces their general applicability. Moreover, the complexity of latent spaces can hinder interpretability and limit parametric representations for complex geometries. Excessive reliance on large databases also raises concerns regarding scalability.

In this work, we introduce a connectivity‐preserving latent diffusion framework for the inverse design of functionally graded mechanical metamaterials with complex spatial property distributions. Unlike existing latent generative approaches that often require heuristic post‐processing to enforce topological consistency, the proposed method integrates discrete graph‐based latent encoding, property‐guided diffusion, and latent interpolation into a unified generative design pipeline, enabling seamless synthesis of graded architectures with structurally consistent interfaces. In contrast to the graph‐based latent approaches discussed above, the present framework explicitly defines and quantifies the transitional connectivity between distinct unit cells and couples the topology‐preserving prior of latent interpolation with property‐guided mechanistic correction, thereby directly addressing the interface‐discontinuity problem that is intrinsic to functionally graded assembly. As schematically illustrated in Figure 1, the framework couples vector‐quantized graph embeddings with a property‐aware latent diffusion process to establish a physically meaningful and connectivity‐preserving design manifold for graded metamaterial generation. By embedding graph‐based unit cell geometries into a vector‐quantized discrete latent space, the framework preserves essential connectivity information while enabling compact representation of diverse microstructural variations. A property‐guided latent diffusion process then iteratively refines latent variables toward target mechanical responses, enabling robust inverse design of new unit cells. Building upon this discrete latent manifold, functionally graded metamaterials are further constructed through latent interpolation and guided diffusion across multiple unit cells, allowing smooth spatial transitions in effective properties without sacrificing structural continuity. Collectively, this hybrid strategy provides a scalable and data‐efficient route for designing mechanically robust FGMs, offering a generalizable foundation for next‐generation architected materials in demanding multifunctional applications.

**FIGURE 1:**
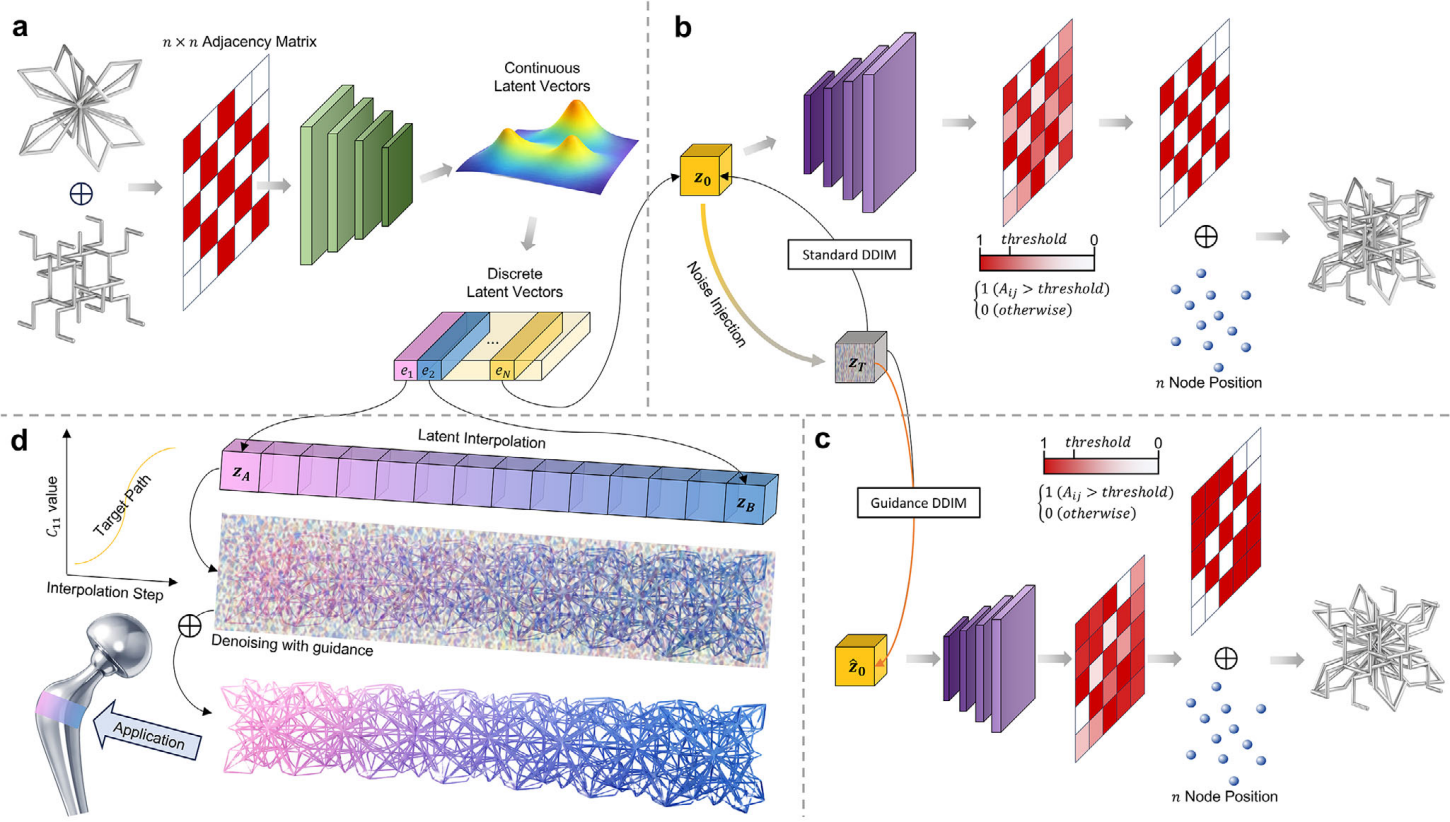
Overview of the proposed latent diffusion–based design framework for metamaterials. (a) Graph‐based unit cell geometries are first represented as adjacency matrices and encoded into a discrete latent space using a vector‐quantized autoencoder, transforming unit cell geometries into compact latent representations while preserving structural intrinsic connectivity. (b) In the standard denoising framework, Gaussian noise is injected into the latent representations, and unit cell geometries are reconstructed through a reverse denoising process. (c) In the mechanistically guided denoising framework, the denoising trajectory is explicitly steered toward regions of the latent space corresponding to target mechanical properties, enabling accurate inverse design. (d) By combining latent interpolation with mechanistically guided denoising, functionally graded metamaterials with spatially continuous variations in microstructure and mechanical properties are constructed. This hybrid strategy enables seamless assembly of graded architectures while maintaining perfect microstructural connectivity.

## 2 Results and Discussion

### 2.1 Mechanistic Guidance Enhances Fidelity of Unit Cell Reconstruction

Figure 2 illustrates the effect of mechanistic guidance on the fidelity of unit cell reconstruction within the latent diffusion process. As shown in Figure 2a, both standard denoising and mechanistic guided diffusion start the reverse generation process from the same noisy latent state *z_T_*, which is obtained through the forward diffusion process. During the reverse process, standard denoising reconstructs unit cell geometries solely based on the learned data distribution, whereas mechanistic guidance denoising explicitly controls the denoising trajectory using property‐based guidance. As a result, the guided denoising process consistently produces reconstructions that more closely resemble the original unit cell geometries in terms of overall geometric morphology.

**FIGURE 2:**
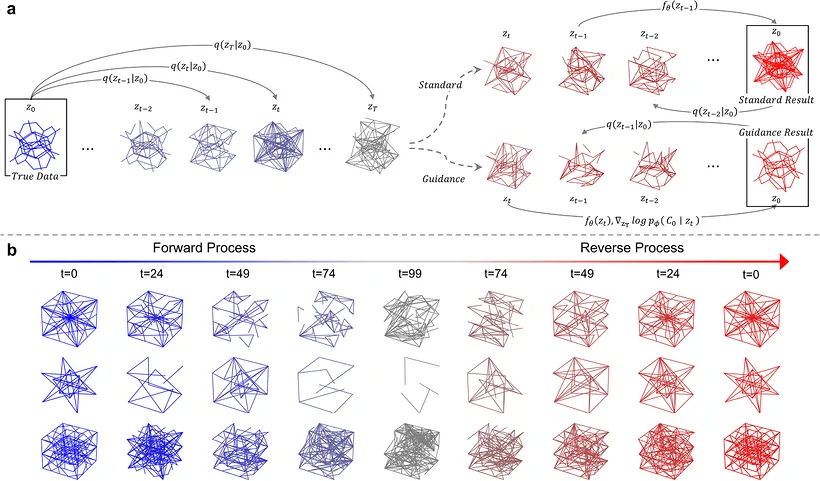
Effect of mechanistic guidance on unit cell reconstruction fidelity in the latent diffusion process. (a) Comparison of reconstruction behavior obtained using standard denoising and mechanistically guided denoising in the latent diffusion process. In the forward process, Gaussian noise is progressively added to the latent representation *z*_0_corresponding to real data, resulting in a noisy state *z_T_*. In the reverse process, noise is removed to reconstruct the latent representation. Compared to standard denoising, mechanistically guided denoising more faithfully reproduces the structural characteristics of the original unit cells. (b) Evolution of latent representations across diffusion time steps. As noise is added in the forward process, structural information is gradually degraded. In the reverse process, mechanistically guided denoising progressively restores structural features from the noisy latent states. A reduced‐step DDIM schedule with (T = 100) was used to balance reconstruction stability and computational cost. The displayed time steps were uniformly sampled along the forward and reverse trajectories to visualize the degradation and recovery of structural information.

This difference becomes more evident in Figure 2b, which visualizes the evolution of latent representations across diffusion timesteps. During the forward process, structural information is progressively degraded as noise is added, ultimately leading to latent representations in which structural features are barely discernible. In contrast, during the reverse process, mechanistic guidance denoising stably and consistently recovers structural features from the noisy latent states, whereas standard denoising exhibits relatively larger deviations from the original structures at intermediate and final stages. Notably, even when the reconstruction task is performed without explicitly specifying a target mechanical property and aims only to recover the original data distribution, the introduction of mechanistic guidance still improves reconstruction accuracy. This suggests that the guidance term plays an effective regularization role in latent space. Overall, these results demonstrate that mechanistic guidance significantly enhances the fidelity of unit cell reconstruction compared to standard diffusion methods, providing a reliable foundation for subsequent inverse design and functionally graded metamaterial generation.

### 2.2 Robustness of Guided Generation Under Varying Noise Levels and Denoising Step Size

To quantitatively evaluate the robustness of the proposed framework, we investigated its behavior by varying two key parameters: (1) the noise level corresponding to different diffusion timesteps, and (2) the denoising step size λ used during guided sampling. These parameters directly affect reconstruction stability in latent space and the fidelity of guided generation and therefore serve as critical indicators of the overall reliability of the model.

First, robustness against different noise conditions was evaluated through reconstruction experiments, in which noisy latent representations generated at various diffusion timesteps were used to recover the original unit cell structures, as shown in Figure 3a. Unit cells with different node densities and combinatorial complexities were selected, such that structural complexity increased with the number of possible configurations. Gaussian noise was then injected into the corresponding latent vectors at different diffusion timesteps, and the resulting noisy latent representations were reconstructed using the proposed model. The results show that the model consistently and stably recovers the original clean unit cell structures across all experimental conditions. Even as the noise level increases or the node density becomes higher, the reconstructed unit cells preserve both geometric morphology and structural connectivity, yielding nearly identical structures. This indicates that the model effectively learns a noise‐invariant mapping from noisy latent representations. *z_t_* to the original latent representation *z*_0_, thereby providing a stable backbone for the latent diffusion framework.

**FIGURE 3:**
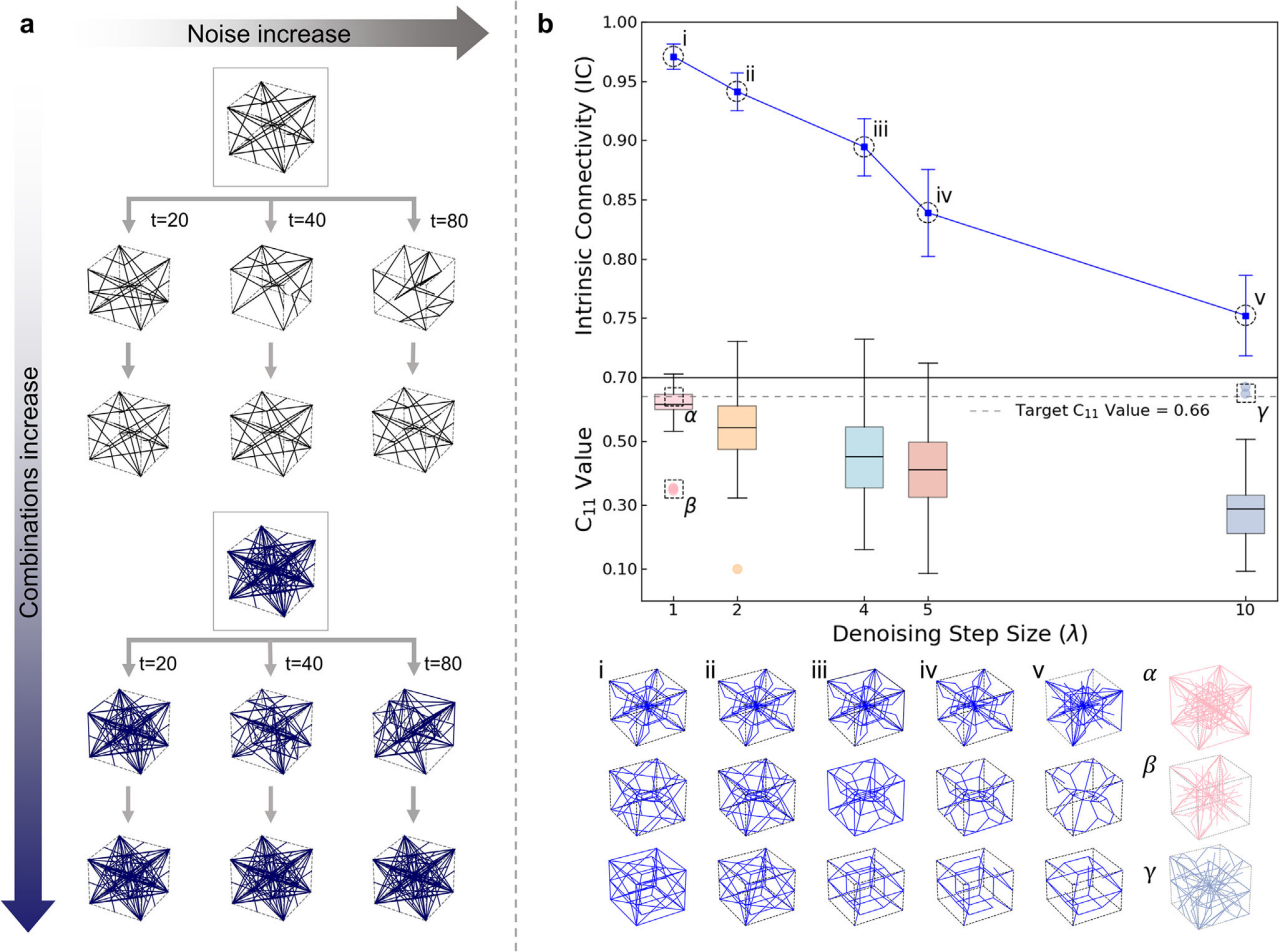
Robustness evaluation of the latent diffusion model with respect to noise level and denoising step size. (a) Unit cell reconstructions obtained by applying diffusion‐based denoising to latent representations at different noise levels (*t*  =  20,  40,  80) under a fixed total diffusion step number *T*  =  100. These noise levels were selected to represent low, intermediate, and high levels of corruption in the latent space. Despite substantial variations in the initial noise magnitude, the geometric morphology and structural connectivity of unit cells are stably recovered in all cases, indicating the high robustness of the proposed latent diffusion framework under diverse noise conditions. (b) Quantitative evaluation of the convergence behavior of intrinsic connectivity and the target elastic constant *C*_11_= 0.66 as a function of the denoising step size λ. The top plot shows the mean IC and standard deviation across different λvalues, while the bottom box plots present the distributions of *C*_11_for generated unit cells. Representative unit cell examples [i–v] correspond to λ = 1, 2, 4, 5 and 10, respectively. The cases labeled α, β, and γ illustrate representative convergence behaviors toward the target *C*_11_: α denotes the closest case to the target at λ = 1, β denotes the largest deviation at λ = 1, and γ denotes the closest case to the target at λ = 10.

Next, we analyzed the effect of the denoising step size λ on the stability of the guided sampling process. Two complementary metrics were used for this evaluation. The first is the intrinsic connectivity (IC), defined in Figure S4a, of the generated unit cell structures, which quantitatively reflects structural degradation caused by incomplete or unstable sampling, particularly in terms of reduced connectivity between neighboring unit cells. The second metric is the convergence toward the target mechanical property, represented by the elastic constant *C*_11_, which measures how accurately guided sampling reproduces the prescribed target property.

Figure 3b presents a quantitative summary of the structural stability and property convergence of guided generation as a function of λ. The top plot shows the mean IC and its standard deviation for different values of λ. As λ increases, the mean connectivity decreases noticeably while the variance increases, indicating reduced sampling stability. In particular, for large λ, connectivity degradation becomes prominent near structural boundaries, implying that skipping too many diffusion steps significantly undermines sampling stability. These results highlight the critical role of denoising step size selection in maintaining structural consistency. The bottom box plots show the distributions of the elastic constant *C*_11_ for unit cells generated with different values of λ. When all diffusion timesteps are fully retraced without skipping (λ  =  1), the generated *C*_11_ distribution exhibits a mean nearly identical to the target value and a very small variance. For λ  =  2, the mean remains close to the target value, but the variance increases. In contrast, for larger step sizes (λ  =  4, 5, 10), the variance increases dramatically, and the mean deviates substantially from the target value. These results clearly demonstrate that the accuracy of guided sampling strongly depends on the denoising step size, and that larger step sizes reduce the fidelity of the denoising process.

### 2.3 Inverse Design of Unseen Unit Cells Toward Target Mechanical Properties

To evaluate the inverse design capability of the proposed framework, we first validated its regression accuracy for mechanical property prediction, followed by an analysis of inverse design results for unit cells that are not included in the training dataset. This two‐step evaluation aims to comprehensively assess whether the model can reliably perform inverse design based on accurate property prediction. Figure 4a compares the regressor performance for predicting the elastic constant *C*_11_ using latent vectors as inputs. The proposed model consistently outperforms conventional machine learning–based regression models, including Random Forest, LightGBM, XGBoost, and MLP. Each point represents a pair of true and predicted values, and the diagonal reference line *y*  =  *x* indicates ideal prediction. While predictions from conventional models are relatively widely scattered around the reference line, the predictions of the proposed model are more tightly clustered, indicating superior predictive precision. These results demonstrate that the model effectively learns the structure–property relationship in latent space, providing a reliable basis for inverse design.

**FIGURE 4:**
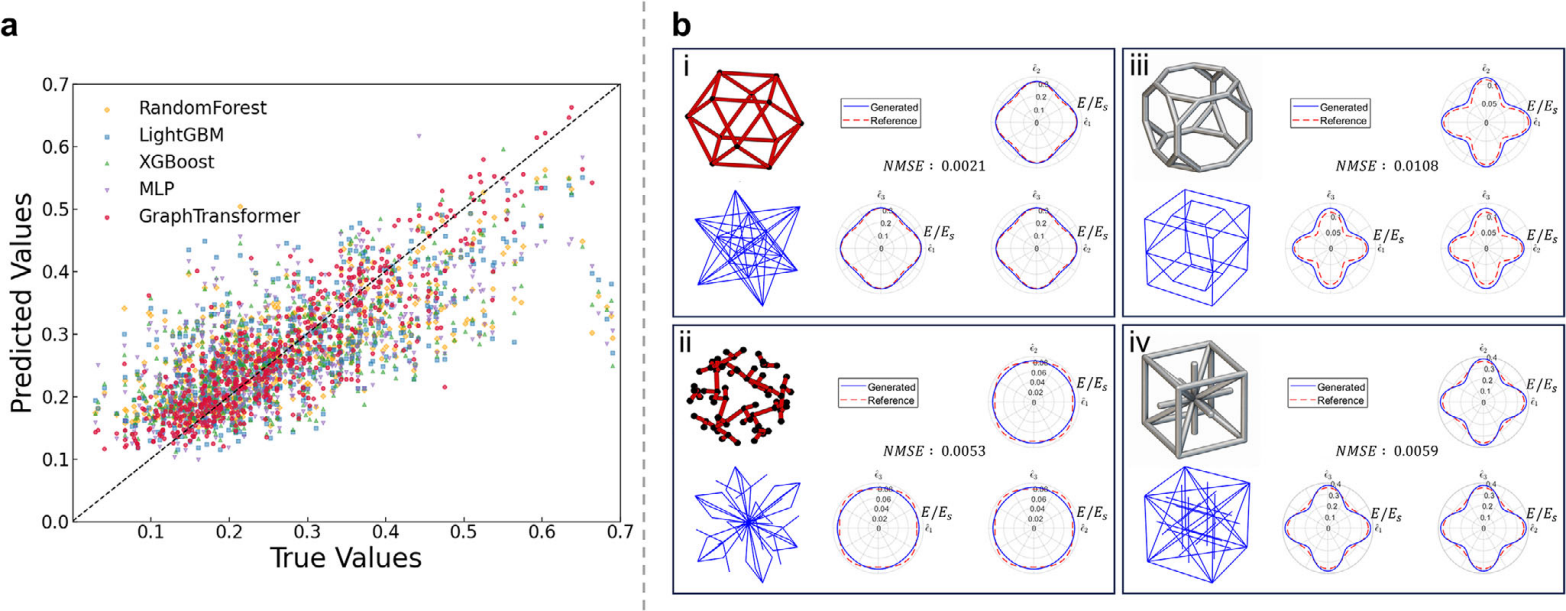
Regression performance and inverse design results for unit cells targeting prescribed mechanical properties. (a) Comparison of *C*_11_prediction performance between a graph transformer–based regression model and conventional machine learning–based regression models (Random Forest, LightGBM, XGBoost, and MLP). Each point represents a pair of true and predicted values, and the diagonal reference line *y*  =  *x*indicates the ideal prediction relationship. A higher concentration of data points near the reference line corresponds to better predictive performance, demonstrating the superior accuracy of the graph transformer–based model. (b) Inverse design results for unit cells not included in the training data distribution, along with their corresponding normalized elastic surfaces compared against prescribed reference responses. Panels (i) and (ii) show inverse design results targeting reference unit cells, while panels (iii) and (iv) present inverse design results targeting well‐known unit cell geometries, namely the truncated cube and the iso‐truss. In all cases, the generated unit cells exhibit strong agreement with the target elastic responses, demonstrating the excellent generalization capability and inverse design accuracy of the proposed framework.

Building on this regression performance, inverse design experiments were conducted to generate unit cell structures that satisfy prescribed target mechanical properties, even when the target structures are not included in the training data distribution. Figure 4b compares the generated unit cell geometries with their corresponding normalized elastic surfaces against the prescribed reference responses. Panels (i) and (ii) show inverse design results targeting reference unit cells reported in the literature [42], while panels (iii) and (iv) present results for targeting well‐known unit cell geometries, namely the truncated cube and the iso‐truss. Importantly, although none of these target structures are included in the training dataset, the generated unit cells reproduce the target elastic responses with high accuracy. The elastic responses are represented as relative elastic surfaces normalized by the Young's modulus of the base material and are visualized through projections onto the *e*_1_  −  *e*_2_, *e*_1_ − *e*_3_ and *e*_2_ − *e*_3_ planes. This representation enables a detailed comparison of directional stiffness characteristics and the degree of agreement with the target responses. To ensure consistency across all evaluations, the strut radius was fixed at 0.05, thereby isolating the effects of topology from those of geometric scaling and enabling a fair assessment of topology‐driven inverse design performance.

### 2.4 Comparison of Interpolation Strategies for Designing Functionally Graded Metamaterials With Perfect Connectivity

Figure 5 compares three interpolation strategies for generating continuous structural transitions between two target unit cell designs: latent interpolation, mechanistic guidance, and the proposed hybrid interpolation. In Figure 5a, latent interpolation produces intermediate structures that maintain high connectivity throughout the transition. However, the corresponding elastic surfaces reveal that the directional stiffness distribution becomes notably anisotropic in the mid‐transition region, suggesting that while the structural topology remains stable, the mechanical response deviates from the intended transition pathway. In Figure 5b, mechanistic guidance generates elastic surfaces in which the stiffness values increase more progressively along the interpolation path. However, the unit cell geometries at several intermediate steps exhibit sparse or disconnected member configurations, and the stiffness distribution also becomes anisotropic. This indicates a degradation of structural integrity due to the absence of sufficient structural constraints. The hybrid interpolation results shown in Figure 5c demonstrate that dense and well‐connected topologies are maintained at all intermediate steps, similar to those produced by latent interpolation. Simultaneously, the elastic surfaces exhibit a gradual and consistent increase in stiffness values from the initial to the final configuration. Furthermore, the elastic surfaces in the mid‐transition region display more circular shapes compared to those of the other two interpolation methods, indicating a more isotropic mechanical response. This near‐isotropic tendency offers advantages in environments subjected to multidirectional loading, providing potential benefits for applications requiring enhanced structural robustness and uniform stiffness behavior.

**FIGURE 5:**
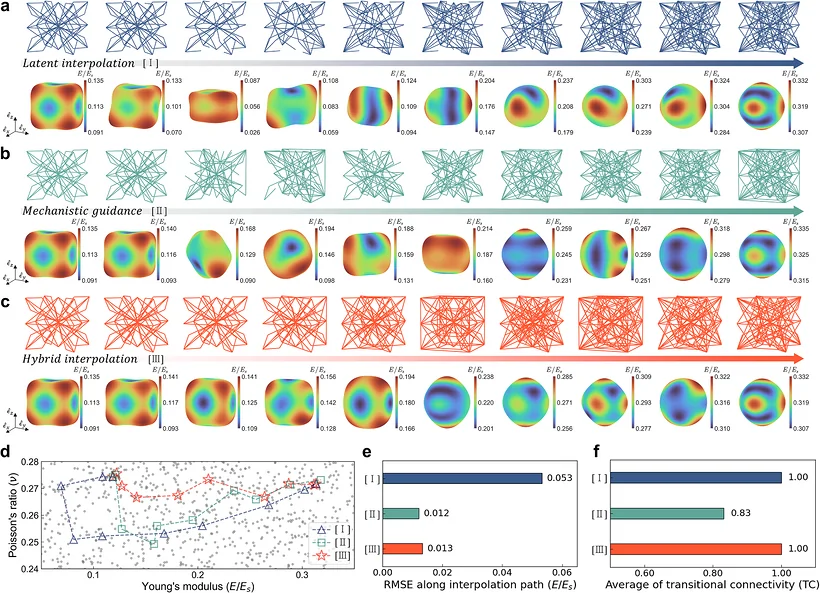
Comparison of interpolation strategies for generating functionally graded unit cell sequences. For all three strategies, the interpolation path between the two endpoint unit cells was divided into the same ten uniformly spaced points, so that the observed differences arise from the interpolation algorithms rather than from differences in sampling positions. (a) Unit cell sequences generated by latent interpolation and their corresponding normalized elastic responses. (b) Unit cell sequences and elastic responses obtained using mechanistic guidance–based interpolation. (c) Unit cell sequences and elastic responses generated by hybrid interpolation combining latent interpolation and mechanistic guidance. For hybrid interpolation, a 30% noise level was applied to the interpolated latent vector, followed by 30 guided denoising steps, enabling property correction while retaining the topology‐preserving initialization. (d) Comparison of trends in the normalized Young's modulus *E*/*E_s_* and Poisson's ratio along the interpolation path for the three interpolation strategies. Each marker denotes one generated unit cell. Because adjacent cells near the path endpoints can have nearly identical normalized Young's modulus and Poisson's ratio values, some markers overlap. (e) Quantitative comparison of stiffness accuracy using the RMSE along the interpolation path in normalized Young's modulus, *E*/*E_s_*. (f) Comparison of the average transitional connectivity and stiffness accuracy across the interpolation steps.

Figure 5d compares the evolution of normalized Young's modulus and Poisson's ratio along the interpolation paths. In the case of latent interpolation, the Young's modulus exhibits non‐monotonic behavior, temporarily decreasing before recovering toward the target value, which indicates insufficient control over the target stiffness. Furthermore, both latent interpolation and mechanistic guidance show regions of abrupt variation in Poisson's ratio compared to hybrid interpolation, suggesting that physically unstable states may arise during the interpolation process. Figure 5e quantitatively evaluates the path‐following accuracy between the target and actual interpolation trajectories using Root Mean Square Error (RMSE). Both hybrid interpolation and mechanistic guidance achieve low error values, confirming that they follow the prescribed stiffness transition path with comparatively high fidelity. Figure 5f compares the interpolation strategies in terms of transitional connectivity (TC), defined in Figure S4b. Mechanistic guidance shows a decline in average TC, reflecting a degradation of structural connectivity during interpolation. In contrast, both latent interpolation and hybrid interpolation maintain consistently high TC values, exhibiting stable transitional behavior. Consequently, hybrid interpolation accurately follows the target interpolation path while simultaneously preserving high TC, demonstrating that it provides the most effective transition route by balancing mechanical accuracy and structural integrity.

These performance differences arise from the distinct roles of structural initialization and property guidance in each strategy. In latent interpolation, the topology of the two endpoint unit cells is propagated through the latent space, allowing the boundary‐node compatibility shared by the endpoints to be largely inherited by the intermediate cells. This explains why TC remains high even when some internal struts become locally sparse. However, because latent interpolation does not directly constrain the stiffness trajectory, the normalized modulus varies non‐monotonically, and the Poisson's ratio can also fluctuate abruptly depending on changes in the internal strut arrangement. In mechanistic guidance, the denoising trajectory is steered toward the target *C*_11_, enabling relatively accurate tracking of the prescribed stiffness path. However, because the guidance objective penalizes only the property error without explicitly enforcing connectivity or the continuity of the Poisson's ratio, intermediate cells can develop weakened boundary connections or irregular internal strut arrangements. As a result, mechanistic guidance can exhibit not only reduced TC but also abrupt variations in the Poisson's ratio.

Therefore, changes in TC and Poisson's ratio can be understood as reflecting different aspects of the generated structures. TC primarily represents interface‐level boundary compatibility, whereas the Poisson's ratio is sensitive to the internal strut arrangement and directional deformation behavior within each unit cell. Hybrid interpolation reconciles these two behaviors by using the interpolated latent vector as a topology‐preserving initialization and then applying mechanistic guidance for property correction. As a result, boundary compatibility and stiffness accuracy are achieved simultaneously, leading to the most balanced performance in terms of stiffness accuracy, transitional connectivity, and mechanical smoothness. Furthermore, the proposed hybrid interpolation promotes smooth transitions in both geometry and homogenized mechanical properties between adjacent unit cells. This continuous structural and property transition reduces abrupt discontinuities at the interfaces, thereby providing an additional advantage in mitigating interface‐related stress concentrations [43].

### 2.5 Application to Femoral Implants With Perfect Connectivity

To validate the practical applicability of the proposed framework, this study applies hybrid interpolation to the design of a functionally graded metamaterial (FGM) for a medical femoral implant, based on a bovine femur sample [44]. The femur exhibits spatially varying mechanical property requirements depending on anatomical location, with stiffness generally increasing from the proximal to the distal region. The objective of this application is to mitigate stress shielding by implementing spatially graded mechanical properties throughout the implant, while simultaneously maintaining structural integrity. This case study is intended as a proof‐of‐concept demonstration of spatial stiffness grading with the proposed connectivity‐preserving framework, rather than a patient‐specific, fully load‐resolved optimization of the implant; accordingly, the elastic constant *C*_11_ is adopted as a representative scalar stiffness descriptor for prescribing and tracking the intended gradient. Figure 6 presents three FGM implant designs generated based on this concept. In the schematic of the femur shown on the left, three color‐coded regions represent distinct design zones with different stiffness requirements, where each color corresponds to a different level of load‐bearing demand at a given anatomical location. Accordingly, a distinct target *C*_11_ path is assigned to each zone, and the target curves defined in this study reflect a transverse property gradient directed from the outer surface of each region toward its center. Notably, the proposed framework enables precise realization of the designer‐intended stiffness gradient path by simultaneously controlling both the location at which property transitions occur and the rate of change.

**FIGURE 6:**
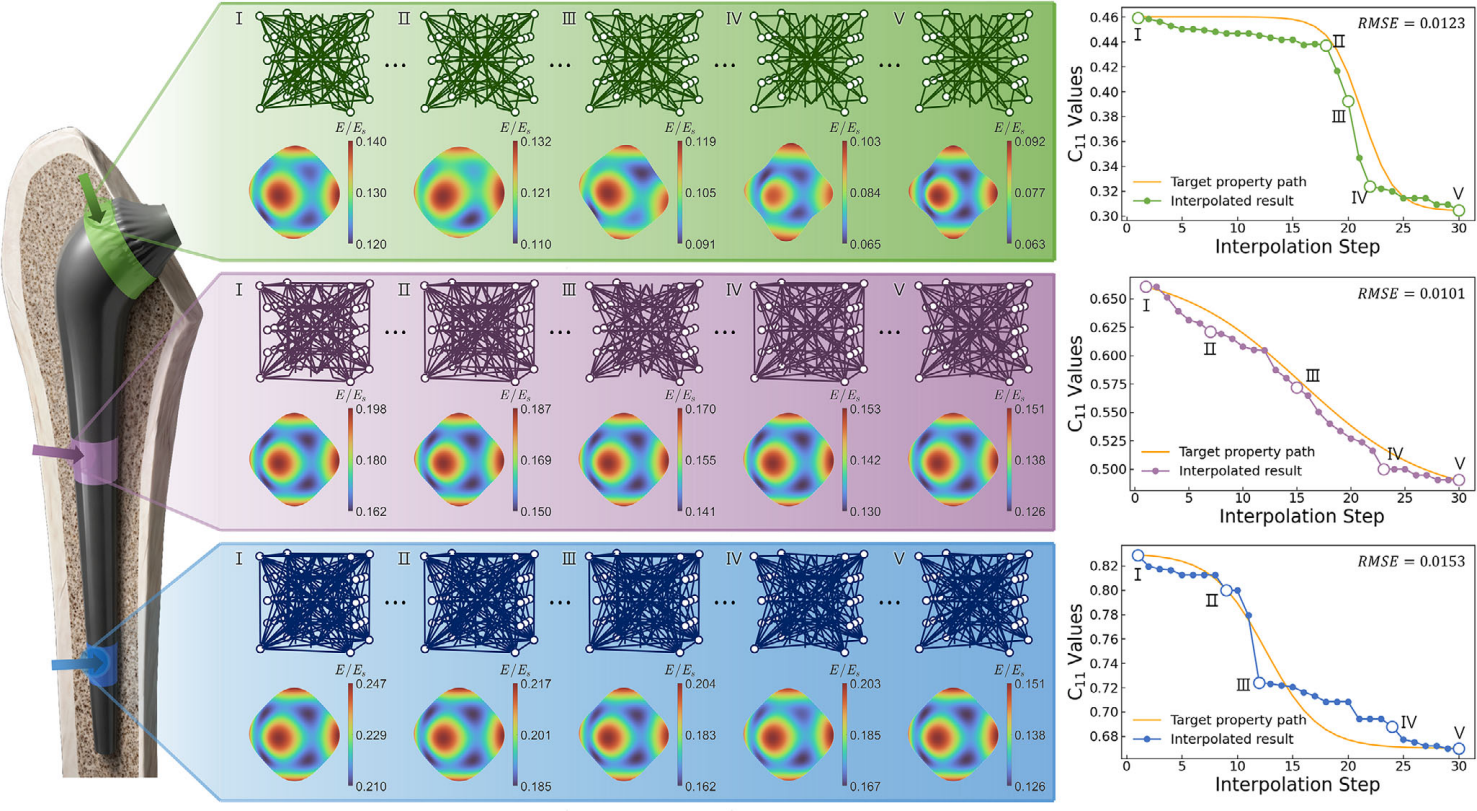
Application to functionally graded metamaterials for a femoral implant. Hybrid interpolation was applied to design spatially graded unit‐cell architectures for the proximal, mid‐shaft, and distal regions of a femoral implant. Representative unit cells (*I–V*) selected at interpolation timestep 30 illustrate the gradual geometric evolution along the target property path, together with the corresponding continuous transition of three‐dimensional Young's modulus distributions. The graph on the right compares the prescribed target *C*_11_ trajectory with the interpolated results, demonstrating that hybrid interpolation follows the target property path while preserving structural connectivity.

Each row in Figure 6 presents five representative unit cells (*I–V*) selected along the property path, illustrating the gradual geometric evolution from the initial to the final unit cell via hybrid interpolation. The 3D Young's modulus surfaces shown beneath each unit cell demonstrate that the elastic response evolves in a continuous and physically consistent manner throughout the transition, without abrupt changes. Since the proposed hybrid interpolation simultaneously maintains structural connectivity and tracks the target property path during transition, designs can be generated without intermediate structural instabilities or geometric discontinuities, even under conditions requiring large stiffness gradients. Furthermore, the generated intermediate structures exhibit elastic responses that are generally close to isotropic, which is advantageous for achieving balanced load‐bearing performance without directional bias. This characteristic can serve as a particularly important design advantage in the femoral implant environment, which is subjected to complex, multi‐directional loading conditions such as walking, torsion, and bending. The graph on the right compares the target *C*_11_paths prescribed for each FGM design with the actual *C*_11_values computed from the generated unit cells. In all three cases, the interpolation results closely follow the target curves with high accuracy and low error, demonstrating that hybrid interpolation can stably reproduce the locally required stiffness distribution in the transverse direction of the implant. Taken together, these results confirm that the hybrid interpolation framework extends beyond a theoretical interpolation technique and can be effectively employed in practical applications such as biomedical implant design, where spatially varying mechanical performance is required. It should be noted that the implant regions experience different loading modes—the proximal region, for instance, is dominated by bending and shear rather than uniaxial compression—so that a fully load‐case‐resolved design would benefit from guiding the generation with shear‐related stiffness components such as *C*_44_, *C*_55_, and *C*_66_, or with the full effective stiffness tensor, instead of the single scalar *C*_11_ used here. Because the homogenization procedure already yields the complete 6 ×  6 tensor, such multi‐component or vector‐valued guidance is a natural extension of the present framework toward region‐specific, load‐aware implant design.

## 3 Conclusion

This study presents a latent diffusion–based design framework for metamaterial design that enables precise control of target mechanical properties while ensuring structural robustness through connectivity‐ preserving design. By formulating the diffusion process in a discrete latent space, the proposed approach allows stable and physically consistent exploration of the design space, overcoming key limitations of conventional data‐driven interpolation methods. The integration of mechanistic guidance into the latent diffusion process plays a critical role in enabling inverse design that directly reflects target mechanical responses. This guidance not only improves convergence toward desired elastic properties but also enhances the overall stability of the diffusion process. The accurate reproduction of elastic responses for target properties outside the training data distribution confirms that the proposed framework captures intrinsic structure–property relationships in the latent space, rather than relying on simple interpolation between known designs. Furthermore, a hybrid interpolation strategy that combines latent‐space interpolation with diffusion‐based guidance is investigated for the design of functionally graded metamaterials. This hybrid approach successfully achieves smooth and controlled transitions in mechanical properties while maintaining complete structural connectivity across the graded sequence, addressing a critical challenge in FGMs design. Finally, the applicability of the proposed framework is demonstrated through its application to medical implant design, where it effectively mitigates stress shielding caused by stiffness mismatch. One limitation concerns the property labels themselves: the effective stiffnesses are obtained from periodic unit‐cell homogenization and therefore represent unit‐cell‐level descriptors rather than the full‐field response of a finite, assembled FGM. Periodic homogenization presumes scale separation and does not explicitly capture the local stress redistribution, finite‐size effects, or neighbouring interactions that arise between heterogeneous adjacent cells, and no corrector term was introduced to compensate for them; within the present framework, the intrinsic and transitional connectivity metrics act to suppress geometric and interface incompatibility between adjacent cells rather than to correct the homogenized stiffness. A quantitative assessment of these neighbouring effects through full‐scale finite element analysis of assembled FGMs, or through corrector‐based homogenization, therefore remains an important direction for future work. These results highlight the practical engineering relevance of the proposed method and suggest its potential for broader applications in mechanistically optimized, functionally graded structures.

## 4 Methods

### 4.1 Database Generation

The unit cell, serving as the building block of the metamaterial, is defined within a cubic design space and modeled as a graph structure comprising connecting edges between 5 × 5 × 5 control nodes distributed uniformly within the domain. Each unit cell is characterized by a node set specifying the positions of the control nodes and an adjacency matrix that defines the connectivity between nodes, the detailed construction of which is described in Supporting Information. Using this approach, 16 independent graph structures were defined as fundamental basis unit cells, effectively capturing diverse graph topologies within the 5 × 5 × 5 design space. These 16 independent basis unit cells facilitated the generation of 2^16^ − 1  =  65, 535 distinct structures through linear combinations. The mechanical properties of each graph‐based mechanical metamaterial were determined using the finite element method combined with the homogenization technique [45], in which each unit cell is treated as a finite‐thickness, voxelized solid and analyzed with 3D continuum finite elements rather than idealized beam or truss members, so that strut thickness and transverse shear deformation are inherently accounted for, as detailed in Supporting Information.

### 4.2 Generative Model Framework

Central to our methodology are deep generative models that leverage latent representations for computational efficiency. The overall training procedure consists of three sequential stages—latent encoding, diffusion‐based reconstruction, and mechanical property estimation—all of which are conducted within a shared latent space. The detailed workflow is illustrated in Figure S3. In the latent encoding stage, the unit cell geometry is embedded into a discrete codebook using a vector‐quantized variational autoencoder (VQ‐VAE) [46]. Building on the traditional VAE, the VQ‐VAE introduces a pivotal modification by adopting a discrete latent space, where the encoder's output is mapped to the nearest vector in a predefined codebook, producing a quantized latent representation. This discrete latent space allows for more structured and interpretable representations compared to continuous latent spaces, making it well‐suited for tasks with inherent discrete structures. The comprehensive mathematical formulation of the VQ‐VAE, including the codebook learning procedure and loss functions, is provided in Supporting Information.

For the diffusion‐based generation, we employ two graph transformer networks that share an identical architectural backbone but differ in output dimensionality, enabling them to perform two distinct tasks: noise prediction and mechanical property estimation. Due to the well‐structured latent space, which intrinsically encodes information about node positions and edge connectivity, the proposed model requires only a latent vector as input to the graph transformer, without explicitly providing node or edge features. The denoising graph transformer is trained to predict a clean latent representation from a noisy input, while the regressor transformer estimates the corresponding mechanical property from the latent vector. The predicted mechanical property then serves as a guidance signal during conditional generation, steering the diffusion process toward regions of latent space that satisfy the target property. The detailed architecture of the graph transformer networks is described in Figure S2.

### 4.3 Denoising Process in Latent Space With Mechanistic Guidance

In this work, we adopt Denoising Diffusion Implicit Models (DDIM) [47], a deterministic formulation of diffusion models derived from ordinary differential equations (ODEs), which enables stable and noise‐free sampling in latent space. The complete mathematical derivation of the forward and reverse diffusion processes, including the marginal and joint distributions, the DDIM deterministic sampling formulation, and the training objective, is provided in Supporting Information.

To enable inverse design toward target mechanical properties, we incorporate mechanistic guidance into the denoising process. Given a target property *C*_0_, the guidance is introduced through a conditional score function defined as:

(1) $$\begin{array}{c} \nabla_{z_{t}}\;\log\left[p_{\theta }\left(z_{t}\right)p_{\phi }\left(C_{0}|z_{t}\right)\right]=\nabla_{z_{t}}\;\log p_{\theta }\left(z_{t}\right)+\nabla_{z_{t}}\log p_{\phi }\left(C_{0}|z_{t}\right) \end{array}$$

where *C*_0_ corresponds to a selected mechanical property from {*C*_11_,*C*_22_,*C*_33_}. $\nabla_{z_{t}}\log p_{\theta }\left(z_{t}\right)$ represents the standard denoising direction of DDIM, which reconstructs the latent variable toward the learned data distribution. $\nabla_{z_{t}}\log p_{\phi }\left(C_{0}|z_{t}\right)$ is introduced as a guidance term that directs the diffusion trajectory toward the latent subspace corresponding to the target property *C*_0_.

The conditional probability $p_{\phi }\left(C_{0}|z_{t}\right)$ is modeled in a Gaussian distribution form as

(2) $$\begin{array}{c} p_{\phi }\;\left(C_{0}|z_{t}\right)=e^{-k{\left(\hat{C}-C_{0}\right)}^{2}} \end{array}$$

where *k* is a tunable coefficient that adjusts the guidance strength, helping to minimize the difference between the predicted property $\hat{C}$ and the target value *C*_0_. This effectively imposes a physical constraint on the data‐driven diffusion process.

Accordingly, the predicted noise term is modified by the guidance component as

(3) $$\begin{array}{c} {\tilde{\varepsilon }}_{t}=\varepsilon_{\theta }^{\left(t\right)}\;\left(z_{t}\right)-\sqrt{1-\alpha_{t}}\nabla_{z_{t}}\log p_{\phi }\left(C_{0}|z_{t}\right) \end{array}$$

where ${\tilde{\varepsilon }}_{t}$ denotes the guidance‐corrected noise estimate produced by the denoising network $\varepsilon_{\theta }^{\left(t\right)}$, and α_*t*_ represents the time‐dependent diffusion coefficient. Through this mechanism, the guidance term continuously refines the latent trajectory during sampling. This shifts the denoising direction toward regions of latent space corresponding to the target mechanical property.

### 4.4 Interpolation Strategies for Functionally Graded Metamaterials

To design functionally graded metamaterials, three interpolation strategies were employed to achieve continuous transitions between graph structures: latent space interpolation, mechanistic guidance interpolation, and a hybrid interpolation combining both methods.

In the latent interpolation approach, two graphs, denoted as A and B, were encoded into their corresponding latent representations *z_A_* and *z_B_*. The interpolation between these vectors was performed using the spherical linear interpolation formulation:

(4) $$\begin{array}{c} \;z_{\lambda }=\frac{\sin\left(\left(1-\lambda \right)\theta \right)}{\sin\left(\theta \right)}\;z_{A}+\frac{\sin\left(\lambda \theta \right)}{\sin\left(\theta \right)}z_{B},\;\lambda \in \left[0,\;1\right] \end{array}$$

where

(5) $$\begin{array}{c} \theta =\arccos\left(\frac{z_{A}\cdot z_{B}}{\left|\left|z_{A}\right|\right|\left|\left|z_{B}\right|\right|}\right) \end{array}$$

This process produced intermediate latent vectors representing a continuous transition between the two graph representations. Each interpolated latent vector was then directly decoded without denoising to reconstruct the corresponding graph structure.

In the mechanistic guidance interpolation, the same interpolation formula is applied to the mechanical properties of graphs *A* and *B*. By interpolating their respective property values, *C_A_* and *C_B_*, a target property *C*_λ_ is obtained, which is then incorporated into the guidance term of the reverse diffusion process to recover a graph structure from its noisy latent representation.

In addition, a hybrid interpolation strategy that combines both approaches was employed. In this procedure, an interpolated latent vector ***z***_λ_ is first generated through latent interpolation, after which a small Gaussian perturbation is added. Mechanistic guidance based on the interpolated target property *C*_λ_ is subsequently applied, thereby integrating property‐conditioned control into the generation process.

## Author Contributions

**Namjung Kim**: Validation, Formal analysis, Writing – review & editing, Supervision, Funding acquisition, Conceptualization. Designed research; **Jongbin Yu**: Writing – original draft, Investigation, Methodology, Validation, Formal analysis, Data curation; **Dosung Lee**: Methodology, Validation;

## Conflicts of Interest

The authors declare no conflicts of interest.

## Supporting information




**Supporting File**: advs76914‐sup‐0001‐SuppMat1.docx.

## Data Availability

The data that support the findings of this study are available from the corresponding author upon reasonable request.
