# Supplementary material for: Latent Diffusion Process With Mechanistic Guidance For Designing Functionally Graded Metamaterials With Perfect Connectivity
Source: Adv Sci (Weinh). 2026 Jul 30:e76914. Online ahead of print. doi: 10.1002/advs.76914 (PMC13423480; doi:10.1002/advs.76914)
Supplement: Supplementary file 1 — Supporting File: advs76914‐sup‐0001‐SuppMat1.docx. [file ADVS-9999-e76914-s001.docx]

**Supporting Information**

**Latent diffusion process with mechanistic guidance for designing functionally graded metamaterials with perfect connectivity**

*Jongbin Yu^1^, Dosung Lee, and Namjung Kim^*,2^*

*^1^Department of Mechanical Engineering, Gachon University, Seongnam, 13120, Korea*

*^2^Department of Mechanical Engineering, Sogang University, Seoul, 04107, Korea*

*Corresponding author

Prof. Namjung Kim: [namjungk@sogang.ac.kr](mailto:namjungk@sogang.ac.kr)

**S1. Metamaterials generation by basis unit cell summation**

Fig. S1 presents a schematic representation of the methodology for constructing both the fundamental geometric structures and the entire database. The unit cell, serving as the building block of repetitive metamaterials, is defined within a cubic design space. This unit cell is modeled as a graph structure comprising connecting edges between $5\times5\times5$ control nodes, distributed uniformly within the design space. Various geometric representations for the unit cell exist, including plate-based ^[1]^, parametric function-based ^[2]^, and cloud-based approaches ^[3]^. In this study, we selected the strut-based representation due to its simplicity and expressive capability. The selection of five control nodes uniformly distributed in each x-, y-, and z-direction was intentional to enable the representation of various well-known unit cells, such as the octet, face-centered cubic, and body-centered cubic, while maintaining computational efficiency. Each unit cell is characterized by a node set, which specifies the positions of the control nodes, and an adjacency matrix, an $N\times N$ square matrix that defines the connectivity between nodes. Using this approach, we defined 16 independent graph structures as fundamental unit cells, effectively capturing diverse graph structures within the $5\times5\times5$ design space.

These 16 independent basis unit cells facilitated the generation of $2^{16}-1 = 65,535$ distinct structures through linear combinations. Notably, the size of our database is 5 to 50 times smaller than those used in recent graph-based unit cell generation studies. This highlights the efficiency of our approach, demonstrating superior performance in unit cell generation despite a significantly reduced database size. The labels, representing the mechanical properties of each graph-based mechanical metamaterial, were determined using the finite element method combined with the homogenization technique, as detailed in the subsequent section.


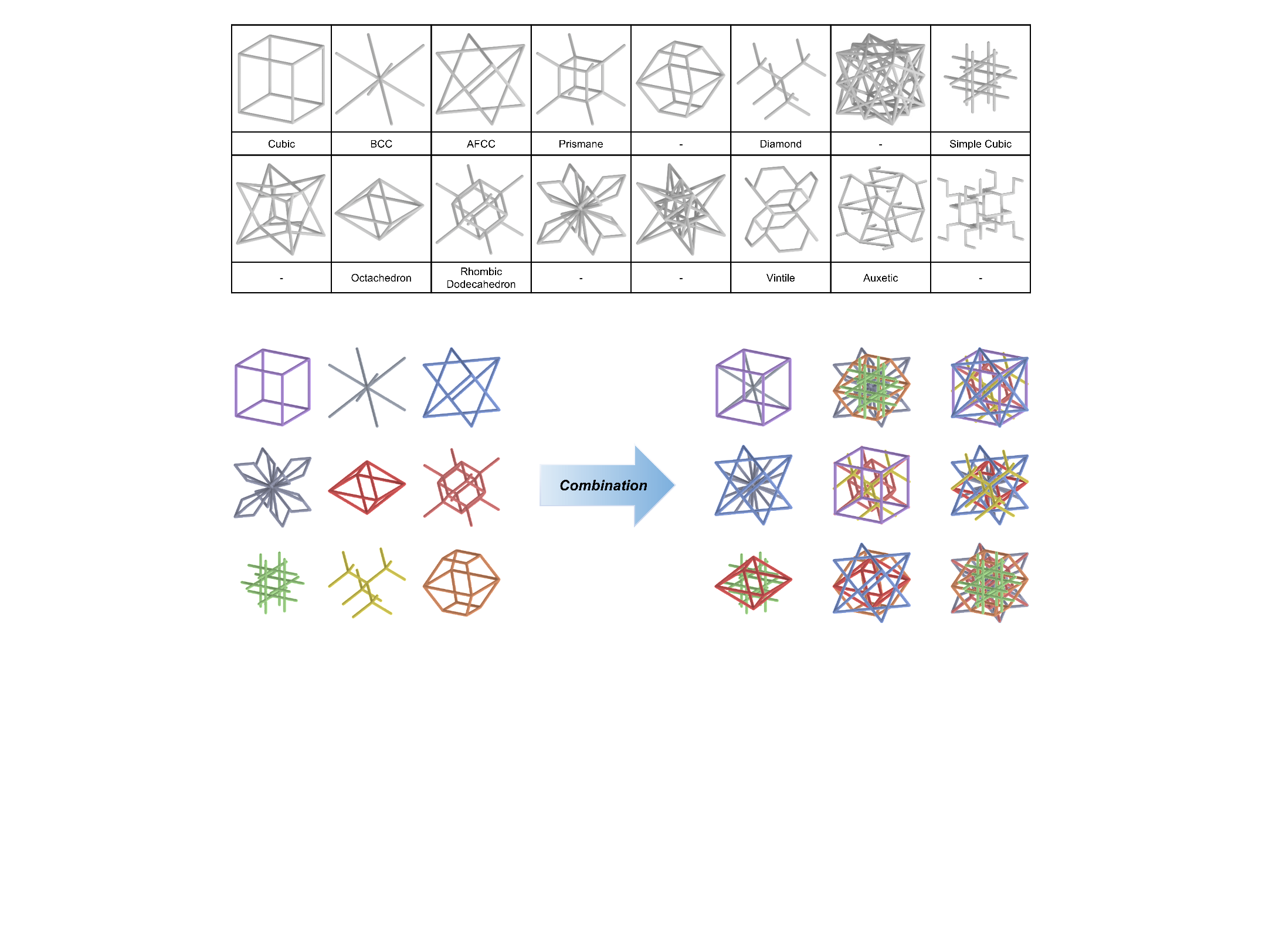


**Figure S1. Basis unit cells and database generation by basis summation.** Sixteen independent basis unit cells defined within a $5\times5\times5$ control-node grid and their linear combinations used to construct the dataset.

**S2. Property estimation by homogenization**

The properties of the unit cell are calculated based on homogenization ^[4,5]^. This approach simplifies the intricate details of complex unit cells by homogenizing them, thereby improving computational efficiency. Each unit cell is treated as an independent periodic representative volume element: every graph edge is dilated about its centerline into a finite-thickness strut by activating all voxels lying within a normalized strut radius of r = 0.05 on a 25 × 25 × 25 grid, and each active voxel is analyzed as an 8-node trilinear hexahedral solid element rather than an idealized beam or truss member, so that strut thickness and transverse shear deformation are represented directly. According to homogenization theory, the difference between $\boldsymbol{\chi}_{\left( \boldsymbol{e} \right)}^{\boldsymbol{0}\left( \boldsymbol{i} \right)}$ and $\boldsymbol{\chi}_{\left( \boldsymbol{e} \right)}^{\left( \boldsymbol{i} \right)}$, along with the element stiffness, is used to calculate the homogenized stiffness tensor $\boldsymbol{C}_{\boldsymbol{ij}}^{\boldsymbol{H}}$​ as described as

$$\begin{aligned} \boldsymbol{C}_{\boldsymbol{ij}}^{\boldsymbol{H}}=\frac{1}{\left| \boldsymbol{V} \right|}\sum_{\left( e \right)} \int_{\boldsymbol{V}_{\boldsymbol{e}}} \left( \boldsymbol{\chi}_{\left( \boldsymbol{e} \right)}^{\boldsymbol{0}\left( \boldsymbol{i} \right)}-\boldsymbol{\chi}_{\left( \boldsymbol{e} \right)}^{\left( \boldsymbol{i} \right)} \right)^{T}\boldsymbol{K}_{\boldsymbol{e}}\left( \boldsymbol{\chi}_{\left( \boldsymbol{e} \right)}^{\boldsymbol{0}\left( \boldsymbol{j} \right)}-\boldsymbol{\chi}_{\left( \boldsymbol{e} \right)}^{\left( \boldsymbol{j} \right)} \right) d\boldsymbol{V}_{\boldsymbol{e}} \end{aligned}$$

where ***V*** represents the total volume of the unit cell, ***V_(e)_*** denotes the volume of the microscopic element, and $\boldsymbol{\chi}_{\left( \boldsymbol{e} \right)}^{\boldsymbol{0}\left( \boldsymbol{i} \right)}$ is the reference displacement field under the unit strain case for the macroscopic strain component ***i***, and $\boldsymbol{\chi}_{\left( \boldsymbol{e} \right)}^{\left( \boldsymbol{i} \right)}$ is the actual displacement field computed from the finite element analysis. The term ***K_(e)_*** denotes the stiffness matrix of the element. The displacement field $\boldsymbol{\chi}_{\boldsymbol{(e)}}^{\boldsymbol{(i)}}$ and $\boldsymbol{\chi}_{\left( \boldsymbol{e} \right)}^{\boldsymbol{0}\left( \boldsymbol{i} \right)}$is obtained as:

$$\begin{aligned} \boldsymbol{\chi}_{\left( \boldsymbol{e} \right)}^{\left( \boldsymbol{i} \right)}\boldsymbol{=}{\boldsymbol{K}_{\left( \boldsymbol{e} \right)}}^{\mathbf{-1}}\boldsymbol{F}_{\left( \boldsymbol{e} \right)}^{\left( \boldsymbol{i} \right)} \end{aligned}$$

where ***K_(e)_*** is the element stiffness matrix, and ***F_(e)_*** is the element load vector. The variable ***i***, ranging from 1 to 6, represents the six distinct types of deformation. $\boldsymbol{\chi}_{\left( \boldsymbol{e} \right)}^{\boldsymbol{0}\left( \boldsymbol{i} \right)}$ represents the value when the macroscopic strain is applied as a unit strain, while $\boldsymbol{\chi}_{\boldsymbol{(e)}}^{\boldsymbol{(i)}}$ is the value obtained from the actual stiffness equation. The element stiffness matrix ***K_(e)_*** is expressed as follows:

$$\begin{aligned} \boldsymbol{K}_{\left( \boldsymbol{e} \right)}=\int_{\boldsymbol{V}_{\left( \boldsymbol{e} \right)}} \boldsymbol{B}^{\boldsymbol{T}}\boldsymbol{C}\boldsymbol{B} d\boldsymbol{V}_{\left( \boldsymbol{e} \right)} \end{aligned}$$

where ***V_(e)_*** is the volume of element, and ***B*** is the strain-displacement matrix, defined through the shape functions of the hexahedron element. Because this operator retains all six strain components ($\varepsilon₁₁$, $\varepsilon₂₂$, $\varepsilon₃₃$, $\gamma₁₂$, $\gamma₂₃$, and $\gamma₁₃$), both axial and transverse shear deformation modes are captured, which is not possible with truss elements that carry only axial force or with beam formulations that may omit transverse shear. The element stiffness matrix ***K_(e)_*** is calculated using Gaussian integration. The element load vector ***F_(e)_*** is calculated as:

$$\begin{aligned} \boldsymbol{F}_{\left( \boldsymbol{e} \right)}^{\left( \boldsymbol{i} \right)}=\int_{\boldsymbol{V}_{\left( \boldsymbol{e} \right)}} \boldsymbol{B}^{\boldsymbol{T}}\boldsymbol{C}\boldsymbol{\epsilon}^{\left( \boldsymbol{i} \right)} d\boldsymbol{V}_{\left( \boldsymbol{e} \right)} \end{aligned}$$

where $\boldsymbol{\epsilon}^{\boldsymbol{i}}$ represents the macroscopic strain, applied as a unit strain.

Because the homogenization described above is performed for each unit cell as an independent periodic representative volume element, the resulting $C^{H}$ values should be interpreted as unit-cell-level effective stiffness descriptors. These descriptors provide consistent mechanical labels for training the property regressor and for evaluating whether the generated unit-cell sequence follows a prescribed stiffness path. However, when heterogeneous unit cells are assembled into a finite functionally graded metamaterial, the local mechanical response may deviate from the independently homogenized bulk properties because of boundary-layer effects, finite-size effects, and mechanical interactions between neighbouring dissimilar cells ^[6,7]^. These neighbouring effects are not explicitly corrected in the present study, and no additional correction coefficient was introduced to modify the homogenized stiffness values.

Therefore, the homogenized stiffness values used in this work are not intended to replace a full-field stress analysis of the assembled FGM structure. Instead, they serve as first-order, unit-cell-level descriptors for generative-model training, inverse design, and stiffness-path evaluation. The intrinsic connectivity (IC) and transitional connectivity (TC) metrics introduced in Section S7 complement this homogenization procedure by quantifying internal and interface-level geometric compatibility. This is particularly relevant because abrupt morphological transitions or insufficient connectivity between adjacent unit cells can induce interface incompatibility and stress concentration in graded cellular structures ^[8]^. However, IC and TC do not constitute a stiffness correction for neighbouring effects. A quantitative assessment of such effects using full-scale finite element simulations of assembled FGMs or corrector-based homogenization remains an important direction for future work.

**S3. Discrete latent space**

The Variational Autoencoder (VAE) framework is designed to establish a stochastic relationship between observed data space, denoted as $x$, and latent space, $z_{e}$. This learning process is interpreted as a directed model characterized by the joint distribution $p_{\theta}\left( x,z \right)=p_{\theta}(x|z_{e})p_{\theta}(z_{e})$ , where $\theta$ represents the model’s parameters, and $p_{\theta}(z_{e})$ is the prior distribution over latent variables. Although the conditioned distribution $p_{\theta}(x|z_{e})$ is parameterized by a decoder, it is generally not tractable. To address this, the VAE employs an encoder, another deep neural network, to map x to z by approximating the posterior distribution. The training’s likelihood function becomes tractable through the evidence lower bound (ELBO) defined as:

$$\begin{aligned} Loss=\mathbb{E}_{q\left( z_{e} | x \right)}\left[ \log p\left( x | z_{e} \right) \right]-\mathbb{D}_{KL}\left[ q(z_{e}|x)||p(z_{e}) \right], \end{aligned}$$

where $\mathbb{D}_{KL}$ stands for the Kullback-Leibler divergence. The loss function of the VAE thus comprises two key components: the reconstruction loss $\mathbb{E}_{q(z_{e}|x)}\left[ \log p(x|z_{e}) \right]$ assessing the model’s reconstruction accuracy, and the regularization loss $\mathbb{D}_{KL}\left[ q(z_{e}|x)||p(z_{e}) \right]$ , which ensures conformity of the learned latent representation to the prior distribution.

Building on the traditional VAE, the vector-quantized (VQ)-VAE introduces a pivotal modification—adopting a discrete latent space ^[9]^. The discrete latent embedding space can be considered as a set of codebooks$\left\{ e_{1}, e_{2}, \ldots e_{N} \right\},$ where each $e_{i}$ is a vector in $R^{D}.$ During the encoding phase, an input $x$ is first mapped to a latent representation $z_{e}(x)$ by the encoder network. The VQ-VAE then assigns $z_{e}(x)$ to the nearest vector in a predefined set, resulting in a quantized latent representation $z_{q}(x)$. This process is mathematically expressed as

$$\begin{aligned} z_{q}\left( x \right)={argmin}_{i}\left| \left| z_{e}\left( x \right)-e_{i} \right| \right|, \end{aligned}$$

where $e_{i}$ denotes the i^th^ vector in the codebook. The reconstruction loss in VQ-VAE, given by $\log p\left( x | z_{q}\left( x \right) \right),$ measures how well the model can reconstruct the input data from this quantized latent representation. Furthermore, the regularization term in VQ-VAE, written as

$$\begin{aligned} \left| \left| sg\left[ z_{e}\left( x \right) \right]-e \right| \right|_{2}^{2}+{\beta\left| \left| z_{e}\left( x \right)-sg\left[ e \right] \right| \right|}_{2}^{2}, \end{aligned}$$

plays a crucial role in learning discrete representation. Here, $sg[\cdot]$ stands for the stop gradient operator. The term ${|\left| z_{e}\left( x \right)-sg[e] \right||}_{2}^{2}$ encourages the encoder’s output to be close to one of the codebook vectors, while ${|\left| sg\left[ z_{e}\left( x \right) \right]-e \right||}_{2}^{2}$ helps maintain the diversity of the codebook vectors. Overall, VQ-VAE’s discrete latent space allows for more structured and interpretable representations compared to the continuous latent space of standard VAEs, making it well-suited for tasks with inherent discrete structures or categories.

**S4. Denoising diffusion probabilistic model in latent space**

Score-based generative diffusion models provide a probabilistic framework for data generation by transforming a data distribution into a noise distribution through a forward diffusion process and subsequently reconstructing it via a reverse denoising process. Among these approaches, the Denoising Diffusion Probabilistic Model (DDPM) ^[10]^ and its deterministic counterpart, the Denoising Diffusion Implicit Model (DDIM) ^[11]^, offer unified score-based formulations grounded in stochastic differential equations (SDEs) and ordinary differential equations (ODEs), respectively.

We define a discrete diffusion process consisting of random variables $z_{t}$ indexed by the time variable $t\in[0,1]$, expressed as $\left\{ z_{t} \right\}_{i=0}^{T}$, where $t=\frac{i}{T}$ and $T$ denotes the total number of time steps. The forward diffusion process is defined with the following marginal and joint distributions. The marginal distribution is expressed as:

$$\begin{aligned} q\left( \mathbf{z}_{t} | \mathbf{z}_{0} \right)\mathcal{=N}\left( \sqrt{\bar{\alpha}_{t}}\mathbf{z}_{0}, \left( 1-\bar{\alpha}_{t} \right)\mathbf{I} \right), \end{aligned}$$

where time dependent coefficients $\alpha_{1:T}$ and $\bar{\alpha}_{t}=\prod_{s=1}^{t} \alpha_{s}$. The joint distribution in DDPM is defined as a Markov chain of conditional transitions,

$$q\left( \mathbf{z}_{1:T} \right|\mathbf{z}_{0})=\prod_{t=1}^{T} q\left( \mathbf{z}_{t} | \mathbf{z}_{t-1} \right).$$

In contrast, DDIM introduces a non-Markovian formulation controlled by a stochasticity parameter $\sigma=(\sigma_{1}, \sigma_{2}, \cdots, \sigma_{T})\in\mathbb{R}_{\geq0}^{T}$, which determines the amount of randomness injected at each step. The joint distribution is defined as

$$\begin{aligned} q_{\sigma}\left( \mathbf{z}_{1:T} | \mathbf{z}_{0} \right)=q_{\sigma}\left( \mathbf{z}_{T} | \mathbf{z}_{0} \right)\prod_{t=2}^{T} q_{\sigma}\left( \mathbf{z}_{t} | \mathbf{z}_{t-1},\mathbf{z}_{0} \right). \end{aligned}$$

To ensure that the marginal distribution $q_{\sigma}\left( \mathbf{z}_{t} | \mathbf{z}_{0} \right)\mathcal{=N(}\sqrt{\bar{\alpha}_{t}}\mathbf{z}_{0}, (1-\bar{\alpha}_{t})\mathbf{I})$ remains consistent for all t, the mean function is defined as

$$\begin{aligned} q_{\sigma}\left( \boldsymbol{z}_{t-1} | \boldsymbol{z}_{t},\boldsymbol{z}_{0} \right)\mathcal{=N}\left( \sqrt{\alpha_{t-1}}\boldsymbol{z}_{0}+\sqrt{1-\alpha_{t-1}-\sigma_{t}^{2}}\frac{\boldsymbol{z}_{t}-\sqrt{\alpha_{t}}\boldsymbol{z}_{0}}{\sqrt{1-\alpha_{t}}},\sigma_{t}^{2}\boldsymbol{I} \right), \end{aligned}$$

where each $\sigma_{t}$ follows the DDIM stochasticity rule:

$$\begin{aligned} \sigma_{t}=\sqrt{\frac{1-\bar{\alpha}_{t-1}}{1-\bar{\alpha}_{t}}}\sqrt{1-\frac{\alpha_{t}}{\alpha_{t-1}}} . \end{aligned}$$

DDIM reformulates the stochastic reverse process of DDPM into a deterministic ODE-based process by eliminating the random noise term, thereby enabling consistent and noise-free sampling. We define a learnable generative process$p_{\theta}(\boldsymbol{z}_{0:T})$, where each conditional transition $p_{\theta}^{\left( t \right)}\left( \boldsymbol{z}_{t-1} \right|\boldsymbol{z}_{t})$ leverages the corresponding reverse conditional distribution $q_{\sigma}\left( \mathbf{z}_{t-1} | \mathbf{z}_{t},\mathbf{z}_{0} \right)$. Given a noisy latent variable $\boldsymbol{z}_{t}$, the denoising network $\boldsymbol{\epsilon}_{\theta}^{\left( t \right)}$ predicts the noise component, and the clean latent representation is estimated as

$$\begin{aligned} \mathbf{z}_{0}\approx\frac{\mathbf{z}_{t}-\sqrt{1-\alpha_{t}}\boldsymbol{\epsilon}_{\theta}^{\left( t \right)}}{\sqrt{\alpha_{t}}} :=f_{\theta}^{\left( t \right)}\left( \mathbf{z}_{t} \right), \end{aligned}$$

where $f_{\theta}^{\left( t \right)}$ denotes the standard $\boldsymbol{z}_{0}$-prediction function derived from denoising output.

The generative process begins with a fixed prior distribution

$$\begin{aligned} p_{\theta}\left( \boldsymbol{z}_{T} \right)\mathcal{\sim N}\left( 0,\boldsymbol{I} \right), \end{aligned}$$

where $\mathcal{N}\left( 0,\boldsymbol{I} \right)$ is a standard normal distribution assuming independent components with unit variance. The process then proceeds recursively as:

$$\begin{aligned} p_{\theta}^{\left( t \right)}\left( z_{t-1} | z_{t} \right)= \left\{ \begin{aligned} \mathcal{N}\left( f_{\theta}^{\left( 1 \right)}\left( z_{1} \right),\sigma_{1}^{2}I \right), if t=1, \\ q_{\sigma}\left( z_{t-1} | z_{t},f_{\theta}^{\left( t \right)}\left( z_{t} \right) \right), otherwise \end{aligned} \right. \end{aligned}$$

to ensure stable generation, Gaussian noise is explicitly added at t = 1.

Each conditional transition is modeled as a Gaussian distribution:

$$\begin{aligned} \boldsymbol{z}_{t-1}=\sqrt{\alpha_{t-1}}\left( \frac{\mathbf{z}_{t}-\sqrt{1-\alpha_{t}}\boldsymbol{\epsilon}_{\theta}^{\left( t \right)}}{\sqrt{\alpha_{t}}} \right) +\sqrt{1-\alpha_{t-1}-\sigma_{t}^{2}}\epsilon_{\theta}^{\left( t \right)}\left( \boldsymbol{z}_{t} \right)+\sigma_{t}\epsilon_{t} \end{aligned}$$

where $\epsilon_{t}$ is random noise. Setting $\sigma_{t}=0$ removes the stochastic term, yielding the deterministic DDIM formulation:

$$\begin{aligned} \boldsymbol{z}_{t-1}=\sqrt{\alpha_{t-1}}f_{\theta}^{\left( t \right)}\left( \boldsymbol{z}_{t} \right)+\sqrt{1-\alpha_{t-1}}\epsilon_{\theta}^{\left( t \right)}\left( \boldsymbol{z}_{t} \right). \end{aligned}$$

Starting from the prior noise $z_{T}\mathcal{\sim N}(0,I)$, the data distribution $p_{\theta}\left( z_{0} \right)$ is reconstructed through iterative reverse transitions in latent space.

Both DDPM and DDIM share the same training objective, as DDIM can be interpreted as a deterministic reformulation of the DDPM framework using the same denoising score matching loss. Assuming all conditional transitions are modeled as Gaussian distributions with learnable mean functions and fixed variances, the objective function is defined as

$$\begin{aligned} \mathcal{L}\left( \theta\right)=\sum_{t=1}^{T} \gamma_{t}\mathbb{E}_{\mathbf{z}_{0}\sim q\left( \boldsymbol{z}_{0} \right),\epsilon_{t}\sim\mathcal{N}\left( \boldsymbol{0}, \boldsymbol{I} \right)}\left[ \left| \left| \epsilon_{\theta}^{\left( t \right)}\left( \sqrt{\alpha_{t}}z_{0}+\sqrt{1-\alpha_{t}}\epsilon_{t} \right)-\epsilon_{t} \right| \right|_{2}^{2} \right] \end{aligned}$$

where $\epsilon_{\theta}:=\left\{ \epsilon_{\theta}^{\left( t \right)} \right\}_{t=1}^{T}$ denotes a collection of $T$ functions, and each $\epsilon_{\theta}^{\left( t \right)}$is parameterized by trainable parameters $\theta^{(t)}$. The weight vector $\gamma:=[\gamma_{1},...,\gamma_{T}]$ consists of positive coefficients that scale the loss at each diffusion step.

**S5. Transformers as a mechanical property estimator and noise predictor**

Our model employs two graph transformer networks that share an identical architectural backbone but differ in output dimensionality, enabling them to perform two distinct tasks: noise prediction and mechanical property estimation. The graph transformer architecture adopted in this work is a modified version of the discrete denoising diffusion model for graph generation (DiGress) ^[12]^, incorporating a self-attention mechanism based on Queries (Q), Keys (K), and Values (V).

Due to the well-structured latent space, which intrinsically encodes information about node positions and edge connectivity, the proposed model requires only a latent vector as input to the graph transformer, without explicitly providing node or edge features. As illustrated in Fig. S2, the denoising graph transformer is trained to predict a denoised latent representation $z_{0}$from a noisy latent vector $z_{t}$, thereby learning a diffusion trajectory that progressively removes noise from the latent space. During this process, the self-attention mechanism enables each element of the latent vector to attend to global contextual information, effectively restoring structural features that have been corrupted by noise.

The regressor transformer takes the refined latent representation as input and quantitatively estimates the corresponding mechanical property of the structure. The predicted mechanical property serves as a guidance signal during conditional generation, steering the diffusion process toward regions of latent space that satisfy the target property. This guidance mechanism facilitates the generation of novel unit cell structures with desired mechanical characteristics.


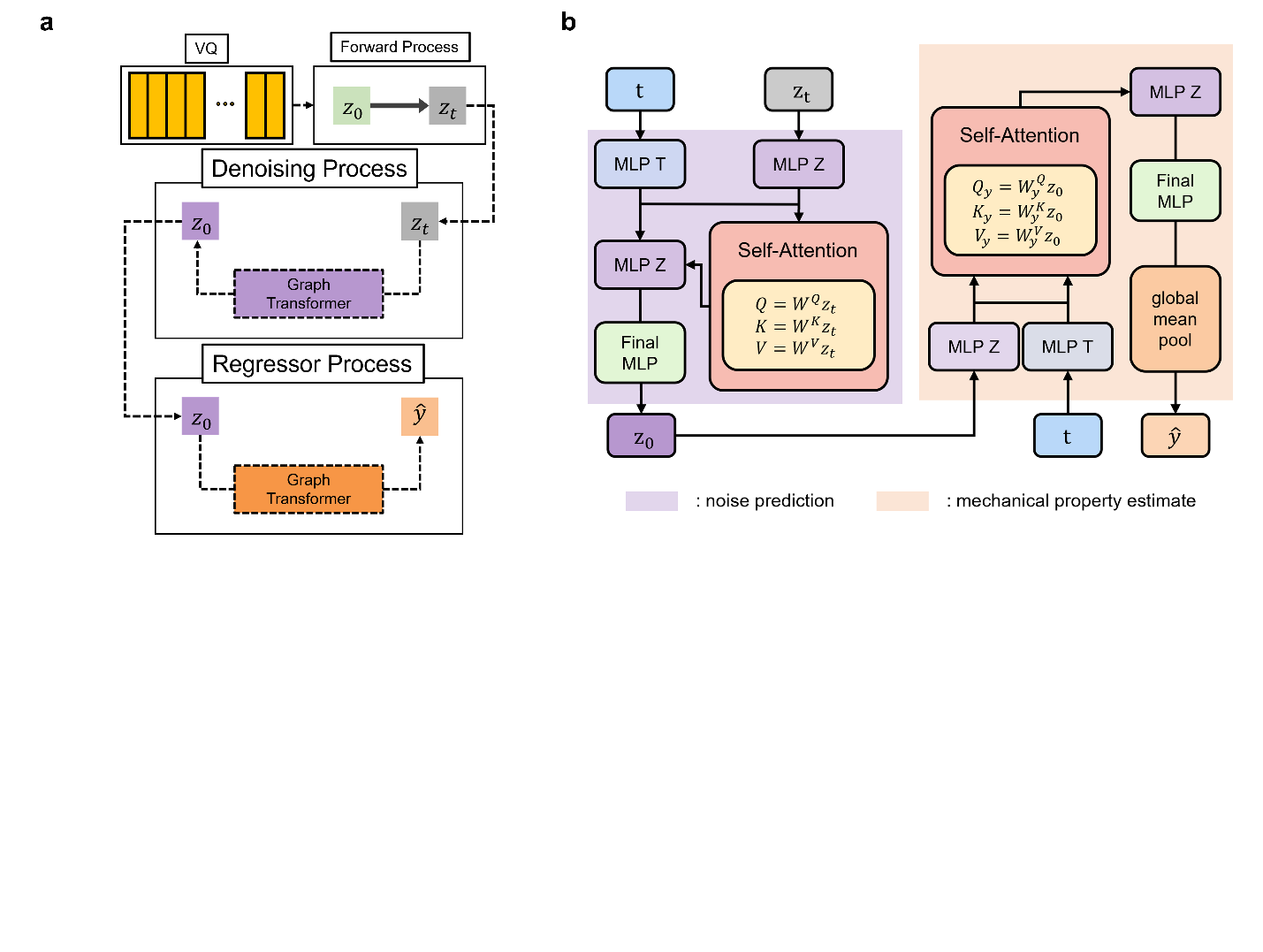


**Figure S2. Schematic architecture of the graph transformer networks.** (a) The denoising transformer predicts the clean latent representation from a noisy input, while the regressor transformer estimates mechanical properties from the latent vector. (b) Architectural overview of the transformer used for mechanical property estimation, where the latent vector is processed through self-attention layers followed by global pooling to produce the predicted property.

**S6. Model architecture**

As shown in Fig. S3, the overall training procedure consists of three sequential stages—latent encoding, diffusion-based reconstruction, and mechanical property estimation—all of which are conducted within a shared latent space. In the latent encoding stage, the unit cell geometry is embedded into a discrete codebook using a VQ-VAE encoder. The encoder, which comprises a Graph Convolutional Network (GCN) followed by fully connected (FC) layers, transforms the input geometry into a low-dimensional latent vector $z_{0}$​, which serves as the input to the subsequent diffusion process.

During the forward diffusion process, Gaussian noise is progressively added to the latent vector $z_{0}$, resulting in a noisy latent representation $z_{t}$. In the DDIM training stage, the model learns the corresponding reverse diffusion process, which reconstructs the original latent vector $\boldsymbol{z}_{0}$ from a noisy latent representation $\boldsymbol{z}_{t}$​. Through this training procedure, the model internalizes the latent-space mappings required to recover structural information from corrupted representations.

In parallel, a regressor training stage is performed using the same latent representation $\boldsymbol{z}_{0}$​ and its associated mechanical property value $y$. In this stage, a separate regressor network is trained to directly predict the mechanical property from the latent vector, enabling quantitative evaluation of mechanical behavior within the latent space.

After training, the model performs conditional generation entirely within the latent space. An initial noisy latent vector $z_{T}$ is sampled from a standard Gaussian distribution, and the trained diffusion model progressively denoises this vector to obtain a refined latent representation. At each diffusion step, the regressor network evaluates the intermediate latent state and computes a guidance score based on the predicted mechanical property. This conditional diffusion process dynamically adjusts the denoising trajectory, steering the generation toward latent regions that satisfy the target property. Finally, the VQ-VAE decoder reconstructs the corresponding unit cell geometry from the refined latent vector $\hat{z}_{0}$, yielding a structure that meets the specified mechanical property requirement.


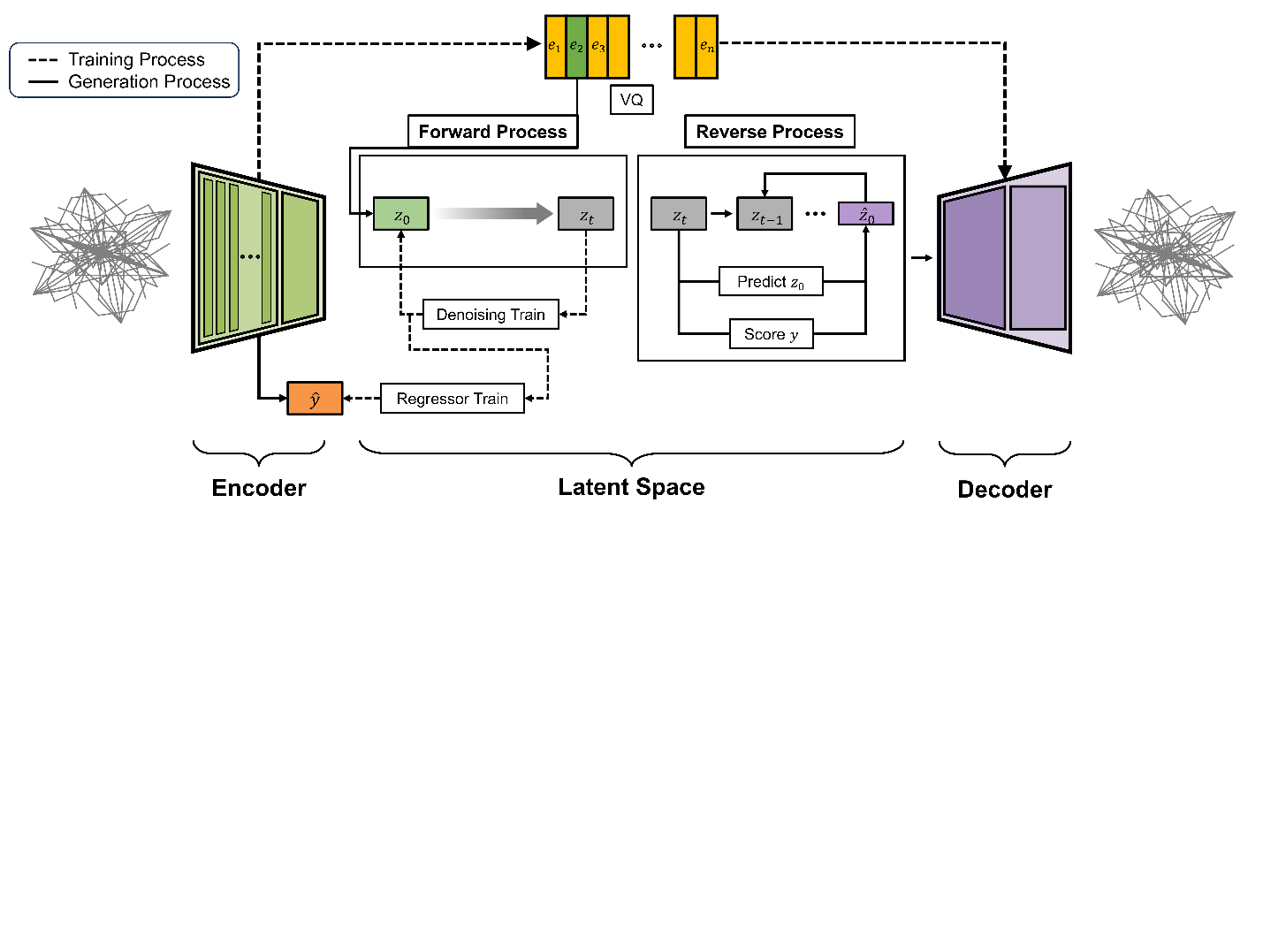


**Figure S3. Overall training and generation workflow of the proposed framework.** The input unit cell geometry is encoded into a discrete latent space and mapped to a codebook-based representation through vector quantization. During training, diffusion-based denoising and mechanical property prediction models are learned from noisy latent representations. During generation, unit cell structures satisfying target mechanical properties are reconstructed from latent vectors through diffusion-based reconstruction with property guidance.

**S7. Connectivity measure**

An essential characteristic of connected 3D unit graphs is their ability to establish connections with multiple neighboring graph cells along the x, y, and z axes. Unconnected nodes at the interfaces present critical challenges, as they can compromise the structural integrity of the assembled units. Moreover, these unconnected nodes often lead to stress concentrations, significantly reducing mechanical properties such as load-bearing capacity and energy absorption. Therefore, quantifying the connectivity between neighboring unit cells is crucial for effectively controlling and optimizing connectivity. To systematically evaluate a graph’s connectivity, we introduced a metric to measure the connectivity of a graph, G.

The first metric, Intrinsic Connectivity (IC), represents the internal connectivity within a single unit graph and is defined as shown in Fig. S4a:

$$\begin{aligned} IC\left( G \right)=\frac{2}{3} \sum_{j\in\left\{ x,y,z \right\}} \frac{\left| P_{j}^{+}\cap P_{j}^{-} \right|}{\left| P_{j}^{+} \right|+\left| P_{j}^{-} \right|} , \end{aligned}$$

where $P_{j}^{+}$represents the subset of nodes are located on the positive boundary face of graph G along the j-th axis, and $P_{j}^{-}$ denotes the subset on the corresponding negative boundary face along the same axis.

In functionally graded metamaterials (FGMs), however, it is also necessary to evaluate the connectivity between adjacent unit cells that differ in geometry. For this purpose, the connectivity between two neighboring graphs$G_{i}$ and $G_{i+1}$ is defined as Transitional Connectivity (TC) as shown in Fig. S4b:

$$\begin{aligned} TC\left( G_{i},G_{i+1} \right)=\frac{\left| P_{i}^{+}\cap P_{i+1}^{-} \right|}{\left| P_{i}^{+} \right|+\left| P_{i+1}^{-} \right|} , \end{aligned}$$

where $P_{i}^{+}$ represents the subset of nodes located on the positive boundary face of graph $G_{i}$ along their connecting direction, and $P_{i+1}^{-}$ represents the subset located on the corresponding negative boundary face of the adjacent graph $G_{i+1}$ along that same direction.

These metrics serve as quantifiable measures, enabling us to ascertain the robustness of the internode connections within the graph and ensure the seamless integration of multiple graph cells in a 3D assembly.


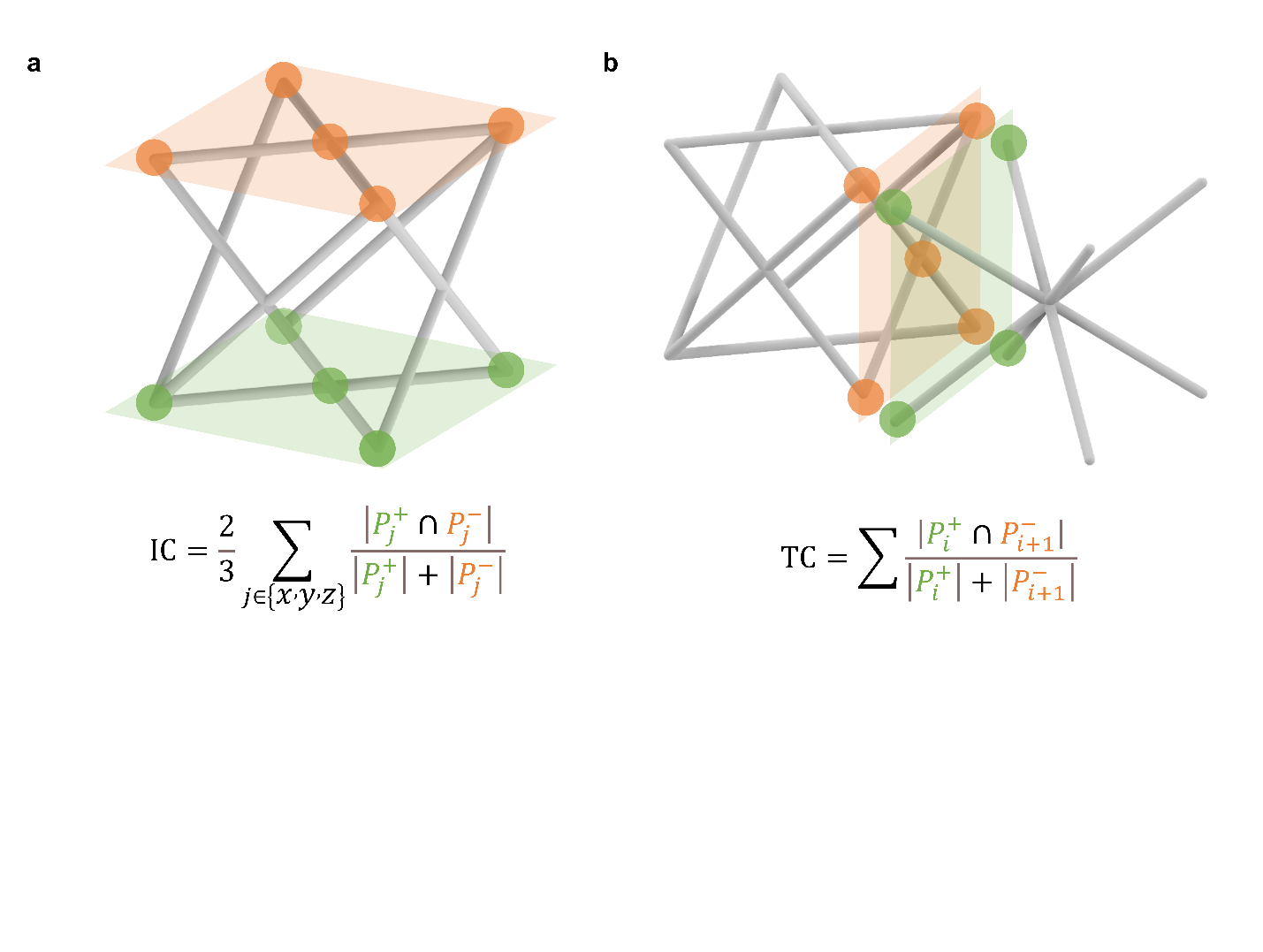


**Figure S4. Definition of connectivity metrics for graph-based unit cells.** (a) Intrinsic Connectivity (IC) measuring internal connectivity within a single unit cell. (b) Transitional Connectivity (TC) quantifying connectivity between adjacent unit cells in a graded assembly.

[1] P. P. Meyer, T. Tancogne-Dejean, D. Mohr, *Acta Mater.* **2024**, *278*, 120246.

[2] A. I. Borovkov, L. B. Maslov, M. A. Zhmaylo, F. D. Tarasenko, L. S. Nezhinskaya, *Materials Physics and Mechanics* **2024**, *52*, 11.

[3] K. Kim, S. Hong, W. Jung, W. Kim, N. Kim, H. Lee, **2025**.

[4] E. Andreassen, C. S. Andreasen, *Comput. Mater. Sci.* **2014**, *83*, 488.

[5] G. Dong, Y. Tang, Y. F. Zhao, *J. Eng. Mater. Technol.* **2019**, *141*.

[6] M. Fergoug, A. Parret-Fréaud, N. Feld, B. Marchand, S. Forest, *Compos. Struct.* **2022**, *285*, 115091.

[7] M. Yoder, L. Thompson, J. Summers, *Int. J. Solids Struct.* **2018**, *143*, 245.

[8] Y. Kim, P. C. H. Nguyen, H. Kim, Y. Choi, *Mater. Des.* **2022**, *218*, 110727.

[9] A. van den Oord, O. Vinyals, K. Kavukcuoglu, **2018**.

[10] J. Ho, A. Jain, P. Abbeel, **2020**.

[11] J. Song, C. Meng, S. Ermon, **2022**.

[12] C. Vignac, I. Krawczuk, A. Siraudin, B. Wang, V. Cevher, P. Frossard, **2023**.
